# Distinct encoding of risk and value in economic choice between multiple risky options^☆^

**DOI:** 10.1016/j.neuroimage.2013.05.023

**Published:** 2013-11-01

**Authors:** Nicholas D. Wright, Mkael Symmonds, Raymond J. Dolan

**Affiliations:** Wellcome Trust Centre for Neuroimaging, Institute of Neurology, University College, London, 12 Queen Square, London WC1N 3BG, UK

**Keywords:** Risk, Loss, fMRI, Approach–avoidance

## Abstract

Neural encoding of value-based stimuli is suggested to involve representations of summary statistics, including risk and expected value (EV). A more complex, but ecologically more common, context is when multiple risky options are evaluated together. However, it is unknown whether encoding related to option evaluation in these situations involves similar principles. Here we employed fMRI during a task that parametrically manipulated EV and risk in two simultaneously presented lotteries, both of which contained either gains or losses. We found representations of EV in medial prefrontal cortex and anterior insula, an encoding that was dependent on which option was chosen (i.e. chosen and unchosen EV) and whether the choice was over gains or losses. Parietal activity reflected whether the riskier or surer option was selected, whilst activity in a network of regions that also included parietal cortex reflected both combined risk and difference in risk for the two options. Our findings provide support for the idea that summary statistics underpin a representation of value-based stimuli, and further that these summary statistics undergo distinct forms of encoding.

## Highlights

• We examine choice between multiple risky options.
• fMRI revealed encoding of value-based stimuli as expected value (EV) and risk.
• Risk and EV underwent distinct types of encoding in distinct brain regions.
• Neural and RT data were also consistent with model-free influences on choice.

## Introduction

Decision-makers frequently have to choose between multiple risky options. For example, animals have to choose between foraging in higher or lower risk patches, or humans whether to invest in higher or lower risk stocks. Such value-based decision-making can be considered within a biologically-grounded, process-based account where a choice evolves from option-evaluation through to action-selection (Corrado et al., 2009). Regarding option-evaluation, recent studies examining the neural basis of risky economic choice have suggested two competing accounts, one that involves a neural representation of outcome distributions by “summary statistics”, such as expected value (EV) and risk (Bossaerts, 2010; Preuschoff et al., 2006; Wright et al., 2012), and another in which subjective value (SV) is determined by the shape of a utility function, with risk-preference emerging as a by-product of that shape (Rangel et al., 2008). Here we seek new evidence for encoding of “summary statistics”, specifically investigating the unknown question of how the summary statistics of multiple, simultaneously evaluated, risky options may be encoded.

We used a task where each trial subject was simultaneously presented with two risky options, one of which had to be selected. Risk is defined here as outcome variance (Bossaerts, 2010). Unlike in a single option, with multiple options there are different ways in which EV and risk may be represented. For both risk and EV we ask whether encoding depends on which option is chosen (i.e. chosen and unchosen EVs; chosen and unchosen risks) or alternatively whether encoding is determined directly by the presented stimuli (e.g. sum or difference in EV or risks). Furthermore, as choices are influenced by whether potential outcomes entail gains or losses (i.e. their valence) (Kahneman and Tversky, 1979) we also asked whether outcome valence differentially affects encoding of EV and risk.

However, even if option-evaluation involved such summary statistics, this does not address how risk, EV or valence influence action-selection. Thus, as a second aim we investigated the choice process from the perspective of a choice architecture in which multiple interacting systems influence action-selection (Dayan, 2008). In model-based systems, stimulus features such as EV, risk or valence may be incorporated within a unified subjective value (SV; utility) computed for each option and where action-selection involves choosing the option with the highest SV. Neurally, we test for encoding of SV. In contrast, in model-free systems that invoke approach–avoidance processes, a key feature is a contingency between stimulus properties and responsive action (i.e. to approach appetitive and to avoid aversive stimulus properties). For both risk and valence we previously found neural and reaction time (RT) data reflecting such contingencies in a task where choices involved a single risky option (Wright et al., 2012), and here asked whether these would be similarly expressed with multiple risky options. A further possibility, in line with choice resulting from multiple interacting systems, would be evidence relating to both: with model-based summary statistic encoding that may influence action-selection through comparator processes and/or approach–avoidance; as well as approach–avoidance to stimulus properties such as valence not requiring model-based processing.

Here we examined the neural basis of risky choice in a task where each trial subjects had to select between two simultaneously presented risky options. Regarding option-evaluation, we hypothesised that there would be encoding of summary statistics representing these options; and were agnostic as to whether these would depend on which option is chosen, or alternatively whether encoding is determined directly by the presented stimuli. Regarding action-selection, we tested for evidence of unified SVs in addition to summary statistics, and for contingencies consistent with approach–avoidance processes.

## Methods

### Participants

All participants, recruited through institutional mailing lists, were healthy and provided informed consent. 25 right-handed participants took part (age mean 24 years, range 19–36; 15 male), with one further participant excluded due to artefacts during fMRI data acquisition. None had taken part in our previous experiments with related tasks (Wright et al., 2012). The University College London Ethics Committee approved the study.

### Task

**Fig. 1:**
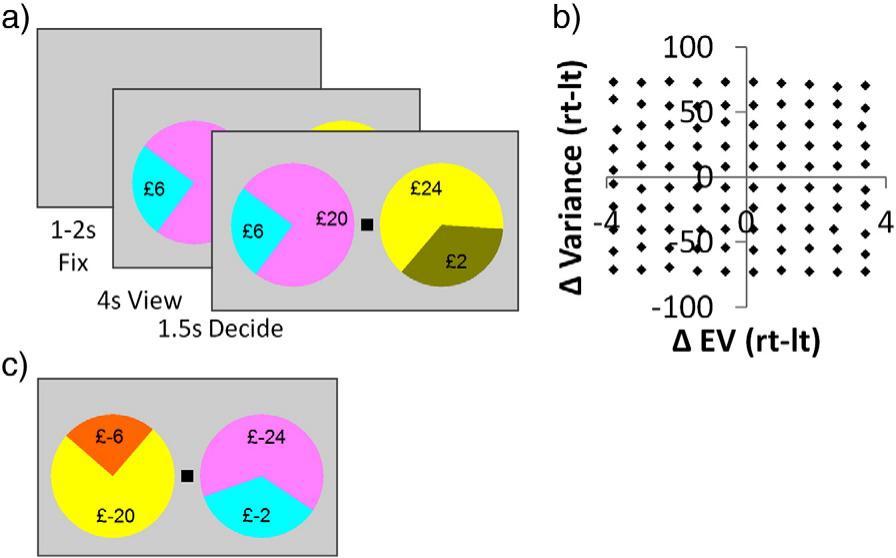
Manipulating risk, expected value and valence. a) In each “gain trial” individuals were presented with two lotteries (each with 2 possible outcomes, both ≥ 0) to consider and select between. They viewed the options for 4 s, after which a black square appeared centrally and they had 1.5 s to input their choice by left or right button press. b) We created set of 100 “gain trials” in which we parametrically and orthogonally manipulated the difference in risk (defined as outcome variance; 10 levels) and EV (10 levels) between the lotteries (i.e. five levels of absolute difference for risk and EV, with these absolute differences used in our analyses). For illustration here we plot each metric for the right minus the left lottery (rt–lt). c) Multiplying all “gain trial” amounts by − 1 gave 100 “loss trials” with identical parametric manipulations. All 200 trials were presented in random order.

The Selection task (Fig. 1) was identical to that used behaviourally in Wright et al. (2012) except that all amounts were doubled for fMRI scanning. There were 200 trials presented in a random order, of which 100 were “gain trials” (all possible outcomes ≥ 0) and 100 were “loss trials” (all outcomes ≤ 0). In each trial, individuals evaluated two lotteries and selected between them. Each trial began with a fixation cross presented for 1–2 s (mean 1.5 s), followed by viewing the options for 4020 ms; and finally a black square appeared to indicate participants had 1500 ms to input their choice by button press (the black square turned white when they chose). If participants did not respond, they received £0 on a “gain trial” and the maximum loss possible on a “loss trial” (£-24).

Our decision-variables of interest were EV, risk and valence. We generated a set of 100 “gain trials” (Fig. 1b and see below), in which we parametrically and orthogonally manipulated the difference in risk (10 levels of variance) and EV (10 levels) between two lotteries (each with two possible outcomes, all ≥ 0), giving five levels of absolute difference for risk and EV (these absolute differences henceforth denoted by ΔVar and ΔEV). To manipulate valence, we simply multiplied all amounts by − 1 to give 100 “loss trials”. This created a set of “gain trials” and a set of “loss trials” that were perfectly matched in their parametric modulations of risk and EV.

Participants began the day with an endowment of £24. After the experiment, one “gain trial” and one “loss trial” were picked at random and their outcomes were added to the endowment to determine final participant payment. Participants could receive between £0–48. There was a low proportion of non-responses (4% ± s.d. 3% of trials). The mean payment received was £23 (range £4–£42).

### Stimulus set

We used the same set of 100 “gain trials” as in Wright et al. (2012) but with all amounts doubled (Fig. 1b). We created this stimulus set in two stages. First, we generated a list of every possible trial within the following constraints: each trial consisted of two pie charts each with two segments; outcomes were between £0 and £24; the smallest allowable probability was 0.1; and the smallest allowable probability increment was 0.05. Second, from within this very large number of potential trials, we selected our set of 100 trials that were the closest match to our desired levels of difference in Var and EV between stimuli. The difference in EV and variance between the options was up to a maximum ΔEV of 3.8 and maximum ΔVar of 73.

For a given lottery with N potential outcomes (*m_1_*, *m_2_*, *… m_N_*), with probabilities *p* = *p_1_*, *p_2_*, *… p_N_*, we define the EV and variance (Var) of the outcome distribution as follows:

(1) $$\mathrm{EV}=\sum_{n=1}^{N}m_{n}p_{n}$$

(2) $$\mathrm{Var}=\sum_{n=1}^{N}{\left(m_{n}-\mathrm{EV}\right)}^{2}p_{n}\text{.}$$

### Statistical analysis

In our behavioural analyses, statistical tests were carried out using paired or independent-samples t-tests, or mixed analyses of variance (ANOVA) in SPSS 17.0 or 20; reported P-values are two-tailed.

### Reaction time analysis

We normalised each individual's RTs by taking the natural logarithm, mean-correcting and dividing by the standard deviation. However, we note that our findings were the same irrespective of having used “raw” or normalised RTs. Regression analysis on participants' RT data was conducted using the glmfit function in Matlab.

### Behavioural modelling

We assessed different utility functions that were identical to those used previously (Wright et al., 2012). We fit data on an individual participant basis. We modelled behaviour by estimating model parameters using maximum likelihood analysis implemented in Matlab. We compared models with different utility functions using Group Bayes Factors, with the Bayesian Information Criterion (BIC) penalising model complexity (Schwarz, 1978). To pre-empt our results we find the same winning model (Mean–Variance–Valence model) as in our previous datasets.

In all our models, on each trial the subjective values, or utilities (*U*), of both options were computed using one of the utility functions below. These values were then compared to generate a trial-by-trial probability of each choice, using a softmax function with a free parameter *β* (constrained between 0 and 10) that allows for noise in action selection.

Initially, we compared three models to ask if behaviour was biassed by risk and valence. First, in a very simple Mean-Only model (*U* = *EV*), individuals only cared about the mean of the options. Second, we asked if choice was also biassed by risk, using a Mean–variance model (*U* = *EV* + *Var* ∗ *ρ*), where risk is measured as variance and *ρ* is a free parameter reflecting an individual's preference for variance (a risk-neutral individual has *ρ* = 0, risk-averse *ρ* < 0, and risk-seeking *ρ* > 0). Third, we asked if both risk and valence bias choice, using a Mean–variance–valence model in which there is a *ρ_gain_* parameter that reflects risk preference in gain trials and a *ρ_loss_* parameter reflecting risk preference in loss trials. We also implemented an Expected Utility model (EUT), Prospect Theory and Cumulative Prospect Theory models, all of which are described in detail in Wright et al. (2012).

### fMRI data acquisition

This was identical to that previously reported in Wright et al. (2012). In a 3 T Allegra scanner (Siemens) each participant underwent one functional run (515 volumes), acquired using a gradient-echo EPI sequence (46 transverse slices; TR, 2.76 s; TE, 30 ms; 3 × 3 mm in-plane resolution; 2 mm slice thickness; 1 mm gap between adjacent slices; z-shim − 0.4 mT/m; positive phase encoding direction; slice tilt − 30°) optimised for OFC and amygdala. We acquired a T1-weighted anatomical scan and local field maps.

### fMRI data analysis

Functional data were analysed using standard procedures in SPM8 (Statistical Parametric Mapping; www.fil.ion.ucl.ac.uk/spm). fMRI time-series were regressed onto a composite general linear model (GLM). The GLM contained boxcars for the length of time the lotteries were displayed (5.5 s) to examine the decision-making process. Delta functions were also included for button presses, lottery onset to account for visual stimulus presentation, and for trials in which subjects failed to respond. In our main GLM, we modelled our neuroimaging data using a 2 valence (gain, loss) by 2 choice (riskier, surer) design. Additional parametric modulators were included, with the height of the boxcar modulated by ΔEV and ΔVariance in each trial. The delta functions and boxcars were convolved with the canonical haemodynamic response function. Movement regressors were included in the GLM. This main GLM design was identical to that used previously in Wright et al. (2012).

We report all activations at P < 0.05 that survive whole brain correction using family-wise error at the cluster level (Friston et al., 1993), unless otherwise stated. Clusters were defined using a threshold of P < 0.005. For presentation, images are displayed at P < 0.001 uncorrected. Unless otherwise stated, small volume correction (P < 0.05) was for a sphere of 10 mm radius around stated coordinates.

#### Alternative parametric designs

We also estimated further GLMs using alternative parametric regressors, described in the Results. Unlike in the basic GLM specified above in which ΔEV and ΔVariance were orthogonal by design, due to a limit in the number of trials this was not possible for all other parametric regressors and there were correlations between some regressors (Table 1). Note that similar correlations for choice-dependent regressors (e.g. chosen SV or unchosen SV) will be contingent on individual participants' choices. Unless otherwise stated, all these models with alternative parametric regressors were based on the same 2 valence (gain, loss) by 2 choice (riskier, surer) design with the same regressors of no interest (e.g. button press). Further, unless otherwise stated all were estimated without orthogonalisation, which enables us to examine only neural activity that correlates with the unique component of each regressor.

## Results

### Choice behaviour

**Fig. 2:**
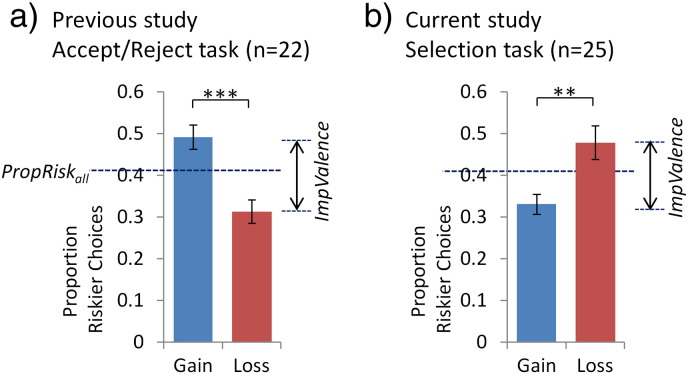
Risk and valence influence choice behaviour. To facilitate direct comparison, we present data from a) our previous fMRI study using the Accept/Reject task (Wright et al., 2012) and b) the current fMRI dataset. The paradigms were carefully matched (e.g. very similar parametric manipulations of ΔEV and ΔVar), except that in the Accept/Reject task in each trial individuals accepted or rejected a lottery relative to a sure option. In both experiments, risk preference is reflected by the proportion of riskier choices made (*PropRisk*; risk-averse < 0.5; risk-neutral = 0.5; risk-seeking > 0.5). Regarding risk, both paradigms revealed the same degree of risk aversion overall (independent t-test *PropRisk_all_* here versus *PropRisk_all_* in previous Accept/Reject fMRI dataset t_(45)_ = 0.07. P > 0.9). Regarding valence, a simple metric for the impact of valence on choice is given by the difference in riskier choices in each domain (*ImpValence* = *PropRisk_gain_* − *PropRisk_loss_*). Individuals were sensitive to valence here, and the magnitude of this valence effect was the same as in our previous fMRI experiment using the Accept/Reject task (independent t-test for modulus of *ImpValence* here versus previous Accept/Reject fMRI dataset t_(45)_ = 0.07. P > 0.7). However, in contrast to the Accept/Reject task, here valence caused the reverse effect such that individuals gambled more for loss compared to gain outcomes. Error bars show s.e.m., **P < 0.005, ***P < 0.00005.

In our Selection task both risk and valence influenced choice, replicating our previous findings (Wright et al., 2012). With respect to risk, a simple metric of risk preference is given by the proportion of riskier choices made overall (*PropRisk*; risk-neutral = 0.5; risk-averse < 0.5; risk-seeking > 0.5). Here, in the Selection task individuals were risk-averse (*PropRisk_all_* 0.40 ± s.d. 0.12; one-sample t-test versus risk-neutral, t_(24)_ = − 3.94, P = 0.0006; Fig. 2). This was similar to our previous fMRI experiment using an Accept/Reject task where individuals accepted or rejected a lottery relative to a sure option (Wright et al., 2012) (independent samples t-test *PropRisk_all_* here versus *PropRisk_all_* in previous Accept/Reject fMRI dataset t_(45)_ = 0.07, P > 0.9).

To examine an influence of valence on choice, a simple metric is given by the difference in riskier choices in each domain (*ImpValence* = *PropRisk_gain_* − *PropRisk_loss_*). We first showed that individuals were sensitive to valence (*ImpValence −* 0.15 ± 0.22; one-sample t-test versus no effect of valence, t_(24)_ = − 3.31, P = 0.003; Fig. 2), and to a similar degree to that seen in our previous fMRI study using the Accept/Reject task (independent samples t-test for modulus of *ImpValence* here versus previous Accept/Reject fMRI dataset t_(45)_ = 0.07. P > 0.7). However, secondly, in contrast to our previous finding in the Accept/Reject task, we now find that valence caused a reverse effect such that individuals gambled more with loss (*PropRisk_loss_* 0.48 ± s.d. 0.20) than gain outcomes (*PropRisk_gain_* 0.33 ± s.d. 0.12; t_(24)_ = − 3.31, P = 0.003). Thus, individuals were risk averse with gains (*PropRisk_gain_* one-sample t-test versus risk-neutral t_(24)_ = − 7.14, P = 2.2 × 10^− 7^) and risk neutral with losses (*PropRisk_loss_* one-sample t-test versus risk-neutral t_(24)_ = − 0.54, P = 0.6).

Finally, we note a considerable heterogeneity between different individuals' preferences related to risk (*PropRisk_all_* mean 0.40, range 0.11 to 0.61) and valence (*ImpValence* mean − 0.15, range − 0.63 to 0.27). In previous datasets an individual's sensitivity to risk and valence were unrelated (Wright et al., 2012) but here these were correlated (*PropRisk_all_* correlated with *ImpValence* r = − 0.48, P = 0.014, d.f. = 23), but here this correlation was not robust to removal of the two participants with the most extreme *PropRisk_all_* (r = − 0.38, P = 0.074, d.f. = 21).

### Behavioural modelling: EV, risk and valence influence choice

Behavioural modelling confirmed that EV, risk and valence influenced choice, with the same winning model here as in our multiple datasets reported previously in Wright et al. (2012). The effects of EV, risk and valence are seen clearly by comparing three related “summary statistic” models. A Mean-Only model where individuals care only about the EV of the options correctly predicted 64% ± s.d. 7% of an individual's choices (summed BIC = 6145). The model is improved by adding the influence of risk in the Mean–Variance model (BIC = 5635), which in turn is greatly improved by also accounting for valence in our Mean–Variance–Valence model (BIC = 5322) where there are separate risk parameters for each valence. This winning Mean–Variance–Valence model outperformed more standard Expected Utility, simple Prospect Theory and more complex Cumulative Prospect Theory models. Furthermore, in absolute terms, the winning Mean–Variance–Valence model correctly predicted 75% ± s.d. 7% of an individual's choices (range 61% to 89%). The risk-related parameters (*ρ*) from this winning model and the simpler metric described above (*PropRisk*) were highly correlated for individuals in the gain trials (r = 0.72, P = 5.5 × 10^− 5^, d.f. = 23) and in the loss trials (r = 0.78, P = 5.0 × 10^− 6^, d.f. = 23). All models have been detailed previously (Wright et al., 2012).

### Reaction time data suggesting risk and valence can influence action-selection through approach–avoidance processes

**Fig. 3:**
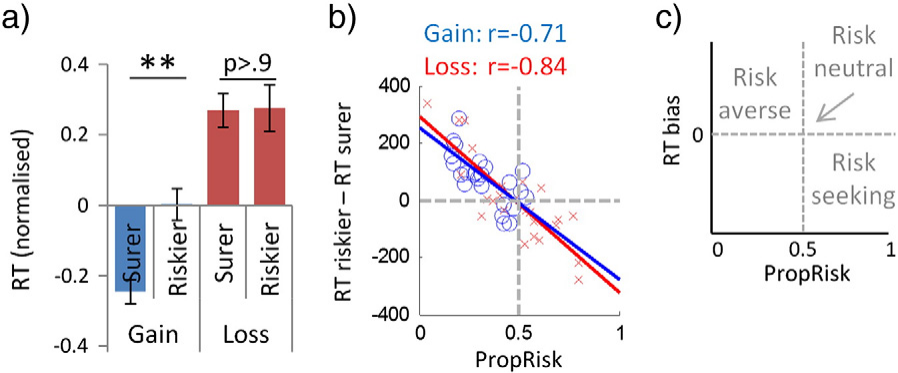
Loss and risk bias reaction times as predicted by approach–avoidance mechanisms. a) Valence biassed RTs as predicted by an approach–avoidance mechanism, with individuals slower to choose (approach) options with losses than with gains. We show the four possible stimulus-action pairs (Gain_riskier_, *Gain_surer_ Loss_riskier_ Loss_surer_*). RTs were normalised per subject. Error bars show s.e.m. b) Risk biassed RTs as predicted by an approach–avoidance mechanism. As the effect of risk depends on individuals' subjective preference we looked between subjects. An individual's risk preference with gains (i.e. *PropRisk_gain_*) strongly predicted their RT bias (*RT_riskier_* − *RT_surer_*) with gains; and their risk preference with losses strongly predicted their RT bias with losses. We observe our predicted pattern, where: risk slowed approach when risk was aversive; risk induced no RT bias when risk was neutral; and risk speeded approach when risk was appetitive (panel c is a cartoon of these predictions). Regression lines are shown, which are not constrained in any way. Grey lines show risk-neutrality in choice (i.e. PropRisk = 0.5) and no RT bias (i.e. RT_riskier_ − RT_surer_ = 0). Gains are in blue, losses in red.

Reaction time (RT) data are consistent with the predictions of a model-free approach–avoidance hypothesis, replicating previous behavioural findings (Wright et al., 2012). Individuals are slower to approach aversive stimuli and are faster to approach appetitive stimuli (Guitart-Masip et al., 2011), which makes simple RT predictions. Regarding valence, individuals will be slower to approach (choose) options containing losses than gains, and this was the case (gains 542 ± 118 msec; losses 654 ± 147; t_(24)_ = 8.17, P < 1 × 10^− 6^). Regarding risk, whether stimulus feature is aversive, neutral or appetitive depends on an individual's risk preference. Thus, we predicted that risk-averse individuals would be significantly slower to approach risk; risk-neutral would show no RT bias and risk-seeking subjects would be faster to approach risk. Note as effects of risk depend on individuals' subjective preferences we examined between subjects. Here, individuals' risk preference with gains (*PropRisk_gain_*) strongly predicted an RT bias when approaching (choosing) the riskier relative to the surer option (RT_riskier_ − RT_surer_) with gains (r = − 0.71, P = 8 × 10^− 5^, d.f. = 23); and risk preference for losses (*PropRisk_loss_*) strongly predicted the RT bias with losses (r = − 0.84, P = < 1 × 10^− 6^, d.f. = 23) (Fig. 3).

Finally, we asked whether there was a facilitatory effect of increased EV on RTs, where we predicted that a larger sum of EVs (∑EV) in a trial would be related to faster RTs. However, we note that as the EV of two options becomes closer (ΔEV) that choice difficulty (and thus RT) will also increase, and that (∑EV) here correlates with variance related stimulus aspects (∑Var, ΔVar; Table 1) which must be accounted for in the analysis. We tested for a facilitatory effect of ∑EV by regressing RTs for each individual subject against a model containing all four regressors (∑EV, ΔEV, ∑Var, ΔVar) to identify correlations between individual's RT data with the unique components of each regressor. Next we brought the regression coefficients from all individuals up to the group level where they are treated as a new response variable (analogous to group analysis in SPM; (Friston, 2004)). As predicted, the group mean regression coefficient for ∑EV was negatively signed (i.e. faster with greater ∑EV), as were those for ΔEV and ΔVar (i.e. slower with smaller differences), and was positively signed for (∑Var) as might be expected given overall risk-aversion. One-sample t-tests showed that these regression coefficients were significantly different to zero (P ≤ 0.003 for all four regressors).

### Factorial fMRI analyses suggesting valence and risk can influence action-selection through approach-avoidance processes

In a simple factorial analysis, for both valence and risk we previously found neural data reflecting contingencies between those stimulus properties and responsive actions in the Accept/Reject task (Wright et al., 2012). As those behavioural contingencies were selectively altered in the current task, we asked if this was reflected neurally by implementing the same 2 valence (gain, loss) by 2 choice (riskier, surer) factorial analysis (details of this main GLM in Methods). Activity is whole-brain cluster-level corrected unless otherwise stated.

Regarding valence there were two main behavioural findings. Firstly, valence influenced choice here to the same degree as in the Accept/Reject task. Secondly, the nature of this valence effect was altered (here increased riskier choices for losses than gains, the reverse was seen in the Accept/Reject task; Fig. 2). Both these effects were reflected neurally. Firstly, consistent with previous data there was enhanced activity for losses > gains in anterior insula (bilateral here: − 33 23 4, Z = 3.7, 89 vox, SVC; 33 26 4, Z = 4.1, 65 vox, SVC), a region implicated in aversive representations (Calder et al., 2001) as well as here also in SMA (whole-brain corrected; Table 2). We again found greater activity for gains > losses in value-related regions including OFC, vmPFC, dorsal and ventral striatum (Table 2), consistent with what we observed previously (greater activity here for gains > losses SVC around the OFC/vmPFC cluster peaks reported previously (Table 1 in Wright et al., 2012), in the striatal cluster reported previously and in ventral striatum for positive stimuli in O'Doherty et al. (2004)).

**Fig. 4:**
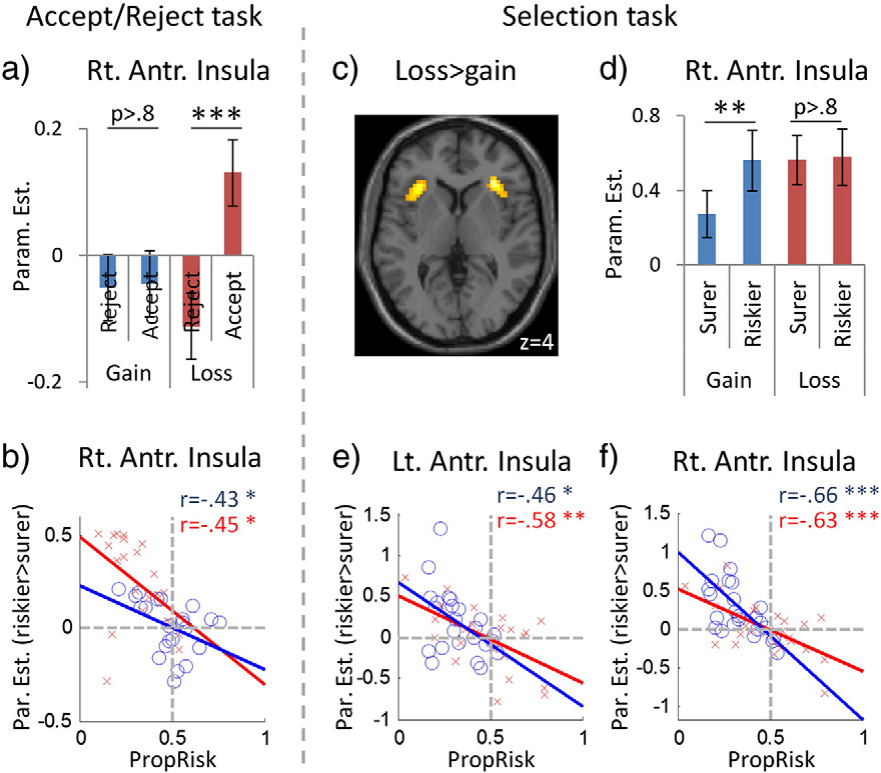
Approaching risk and loss: neural data. Valence and risk may affect action-selection by influencing the disposition to approach economic stimuli, as suggested by fMRI data from the Accept/Reject task (previously reported in Wright et al., 2012) (panels a-b) and new data from the Selection task (panels c-f). a) Regarding valence, in the Accept/Reject task bilateral anterior insula/IFG showed increased activity when approaching (choosing) the lottery with losses (*Loss_risky_*), the specific stimulus-action pair to which individuals were most averse of the four possible in the task (*Gain_risky_*, *Gain_sure_ Loss_risky_ Loss_sure_*) (data from the peak for the choice-by-valence interaction in that task, 30 26–8). However, in the Selection task during loss trials individuals had to approach a lottery with losses, and now bilateral anterior insula activity was raised equally for both options — shown by the main effect for losses > gains (panel c) and no difference in activity between choosing riskier or surer option with losses (panel d, taken from the peak for losses > gains here). Regarding risk, the more averse an individual was to risk (i.e. lower *PropRisk*), the greater the activity when approaching riskier relative to the surer option, in the gain trials (*PropRisk_gain_* vs. riskier > surer in gains, plotted in blue) and loss trials (*PropRisk_loss_* and riskier > surer in losses, in red). In anterior insula for contrasts across subjects that were distinct from the contrasts on which the between subjects plot is based (Accept/Reject peak for choice by valence interaction in panel b; Selection task peak for losses > gains in panels e and f). Error bars indicate s.e.m.. *<0.05, **<0.005, ***<0.001.

We next asked whether the second finding of the altered nature of the valence effect between tasks was also mirrored at the neural level. In the Accept/Reject task the least preferred valence-action pair behaviourally (*Loss_risky_*; Fig. 2a here) was the only one associated with increased anterior insula activity (Fig. 4a here). In contrast, behaviour suggested that in the current study there was equal aversiveness in both actions with losses (i.e. risk-neutral choices and equally slowed RTs with losses here; Fig. 2b), and this was reflected here in a similar degree of enhanced bilateral anterior insula and SMA activity when approaching either option with losses (Fig. 4d; no activity for *Loss_riskier_* > *Loss_surer_* or the reverse in 10 mm spheres centred on the peaks in these regions for losses > gains here or for the interaction in the Accept/Reject task). Further, this did not simply reflect a lack of choice-related activity in these regions, as behaviour across subjects showed that aversion to risk with gains was reflected neurally by increased activity for choosing the riskier than surer option with gains (i.e. *Gain_riskier_* > *Gain_surer_*) in right AI and SMA (10 mm spheres as above, SVC; Fig. 4).

Regarding risk, across subjects there was equivalent aversion to risk overall (i.e. collapsed across valence) as in our previous task (Fig. 2). This similarity was reflected neurally, as we again observed increased activity for approaching (choosing) the riskier, relative to the surer, option in multiple regions (right parietal cortex, mid-cingulate/dmPFC, right anterior insula/IFG) but nothing in the reverse contrast (i.e. chose surer > riskier; Table 2). Furthermore, consistent with an approach–avoidance hypothesis, where here as previously the more averse an individual was to risk (i.e. lower *PropRisk_all_*) the greater the activity evoked when approaching (choosing) the riskier option in bilateral anterior insula, dmPFC/SMA, bilateral parietal cortex and thalamus/caudate (Fig. 4). Analysing each valence separately showed similarly asymmetric patterns across and between subjects (Supplementary results).

Finally, we asked if these findings were specific to valence and risk, or were instead related more generally to choosing options containing less preferred stimulus aspects (e.g. lower EV or lower SV). No such activity was seen either in an alternative factorial design using EV to define choice (i.e. 2 valence [gain, loss] by 2 choice [higher EV, lower EV]), or in a further alternative GLM that used subject-specific SVs from the winning behavioural model to define choice (i.e. chose higher SV or lower SV). Further, our findings related to approaching risk and loss above were still evident even after removing activity related to choosing either lower/higher EV or lower/higher SV (details in Supplementary materials).

### Parametric analyses of EV and variance encoding

We next used parametric analyses to examine EV and risk encoding in multiple risky options. The difference in EV and risk between the two options in each trial (ΔEV and ΔVar) were used as parametric modulators in the main GLM above, and corresponded to the manipulations of ΔEV and ΔVar in the stimulus set (Fig. 1b). In our previous Accept/Reject task the parametric regressors for EV and risk also represented ΔEV and ΔVar (Wright et al., 2012). As previously there was a positive correlation with ΔVar, although here in left anterior insula and medial PFC rather than in posterior parietal cortex seen before, and again no correlation with EV (Table S2).

However, a problem for any paradigm in which choices involve only one risky option (including our previous study) is that a ΔVar regressor is identical to a regressor reflecting the sum or mean of the risk (∑Var). Such activity may be predicted for a region simultaneously encoding both options. If a fixed sure option is used between trials, regressors for ΔEV and ∑EV are also identical. Importantly, in the current task with two risky options these are not identical. Indeed a modified GLM instead using ∑EV and ∑Var revealed more widespread risk-related activity with ∑Var, that included bilateral posterior parietal cortex consistent with our previous study (and also dmPFC, bilateral anterior insula, bilateral dorsal striatum, left MTG, left MFG; no correlation with ∑EV; Table S2).

However, although regions correlating with ∑Var and ΔVar in these separate GLMs only partially overlapped, these regressors were correlated (Table 1). Thus, to identify an activity uniquely attributable to each regressor (i.e. not with their shared components) in a new GLM we included all four parametric regressors without orthogonalisation (∑EV, ΔEV, ∑Var, ΔVar). Here the activity strongly correlated with the unique components of ∑Var (positive correlation in left posterior parietal cortex, left and right IFG/MFG, cerebellum and right sensorimotor cortex) and ΔVar (positive correlation in ventromedial PFC/subgenual ACC; and negative correlation in right and left MFG and occipito-temporal cortex; Table 3). No activity correlated with ∑EV or ΔEV.

Whilst EV encoding was not revealed by these models an alternative is that EV encoding is determined by which option is chosen. Such choice-determined encoding has been suggested for SV (FitzGerald et al., 2009), and is shown for risk in the factorial analyses above where greater activity is seen when the chosen option is riskier than the surer option (Table 2). We tested for such choice-determined encoding of EV, and also asked if parametric analysis revealed additional choice-determined activity for risk, in a new GLM with four regressors: EV_chosen_, EV_unchosen_, Var_chosen_, Var_unchosen_. For risk, the only additional choice-determined activity revealed by this parametric analysis was a positive correlation with the unique component of Var_chosen_ in right posterior parietal cortex, an area also seen in the stimulus-determined model above (peak coordinates for Var_chosen_ also showed activity for ∑Var in the stimulus-determined model). However, as predicted, for EV the choice-determined model revealed extensive activity, and furthermore this was in the directions to be expected for chosen and unchosen values. The unique component of EV_chosen_ positively correlated with activity in precuneus (whole brain corrected) and in OFC previously strongly associated with value (3 44–20, Z = 4.0, 55 vox, SVC), and no regions negatively correlated with EV_chosen_. The unique component of EV_unchosen_ negatively correlated with activity in left premotor cortex, hippocampus and cerebellum (whole brain corrected) and OFC (− 6 44–14 Z = 3.5, 64 vox, SVC), and no regions positively correlated.

Finally, we asked if EV and risk encoding differs when potential outcomes entail gains or losses. In the choice-determined model immediately above that revealed EV-related activity (i.e. with EV_chosen_, EV_unchosen_, Var_chosen_, Var_unchosen_), we also observed extensive activity for the interaction of encoding in gains versus encoding in losses with both EV_chosen_ and EV_unchosen_ (Table 3; Table S3 reports a similar pattern in the stimulus-determined model). Further, this effect of valence on EV encoding was strikingly asymmetric: the chosen option was more positively correlated in gains than losses (EV_chosen_ in gains > EV_chosen_ in losses: dmPFC/ACC, left anterior insula/IFG, left posterior parietal and bilateral MTG) with nothing for the reverse; whilst the unchosen option was more positively correlated in losses than gains (EV_unchosen_ in losses > EV_unchosen_ in gains: pre-SMA/SMA/MCC, right anterior insula/IFG and right posterior parietal) with nothing seen for the reverse. In both cases the interaction resulted from divergent effects in both gains and losses, with activity for EV_chosen_ at the highest peak in each region driven by a positive correlation with gains and negative with losses, with the reverse pattern at the peaks seen for EV_unchosen_. Note that with gains there is a positive correlation with chosen EV (e.g. higher for an EV of £14 than £10; where £10 is the lower EV and has lower activity), and with losses there is a negative correlation with chosen EV (e.g. higher with £-14 than £-10; where £-14 is the lower EV and has higher activity), so activity here does not just positively correlate with chosen EV. In contrast, for risk there was no interaction between encoding in gains versus encoding in losses for any of the risk-related regressors, in the stimulus-determined (∑Var, ΔVar) or choice-determined (Var_chosen,_ Var_unchosen_) models. We observe correlations with ∑Var in both gains (left IFG, left caudate) and losses (incl. right MFG/IFG, left IFG) and a conjunction between activity correlating with ∑Var in both gains and losses (positive correlation in left IFG pars. triang. and operc., − 48 44–8, Z = 3.78, 62 vox, SVC P = 0.007).

### Subjective value

Finally, we tested for encoding of subject-specific SVs from our winning behavioural model in both a choice-determined model (with parametric regressors of SV_chosen_ and SV_unchosen_) and a stimulus-determined model (with ∑SV, ΔSV). The choice-determined model revealed extensive activity, and furthermore this was in the directions to be expected for chosen and unchosen values (Table S4). The unique component of SV_chosen_ positively correlated with activity in SMA, posterior insula/operculum and bilateral sensorimotor cortex, and no regions negatively correlated with SV_chosen_. The unique component of SV_unchosen_ negatively correlated with activity in OFC, right amygdala and left hippocampus, and no regions positively correlated. In contrast, in the stimulus-determined model there were no correlations with either ∑SV or ΔSV.

We can also ask if these SV_chosen_ and SV_unchosen_ regressors better capture activity than EV_chosen_ and EV_unchosen_ in a comparable model containing only those two regressors. These models with SV and EV models revealed similar regions (Table S4), although interestingly the negative correlation with SV_unchosen_ in OFC and right amygdala survived whole brain correction, whilst activity for EV_unchosen_ in these same areas was neither as widespread nor significant (Fig. S1).

## Discussion

Value-based decision-making can be considered within a process-based account of choice that evolves from option-evaluation to action-selection (Corrado et al., 2009). Regarding option-evaluation, studies examining the neural basis of risky economic choice have suggested two main competing accounts: one involves “summary statistics” that describe the distribution of possible outcomes from a risky choice (Bossaerts, 2010; Preuschoff et al., 2006); the other posits a subjective value (SV) determined by the shape of a utility function, with risk-preference emerging as a by-product of that shape (Rangel et al., 2008). Here we provide further evidence for encoding of “summary statistics”, and by using multiple risky options and manipulating valence we highlight a new characterisation of such encoding. In contrast to these data, there was no clear evidence for encoding of SV in addition to EV.

Risk encoding was seen for both ∑Var and ΔVar in distinct neural regions. These two risk-related metrics are identical when a choice involves only one risky option, as in our previous work (Wright et al., 2012). Activity related to ∑Var might be expected in regions simultaneously encoding risk in both options, and here we observed correlations with the unique component of ∑Var in regions that included parietal cortex. Such parietal activity replicates our previous data with a single risky option in the Accept/Reject task (Wright et al., 2012), and concurs with single unit and fMRI data showing enhanced activity in a similar region during risky decision-making (Huettel et al., 2005; Mohr et al., 2010; Platt and Glimcher, 1999). The difference in risk between options (ΔVar) correlated with activity in vmPFC, a region strongly associated with value difference (Rushworth et al., 2011), although we note this may not simply reflect a subjective value representation as this would also be expected to differ between individuals depending on their individual risk-preference. In addition to demonstrating this risk encoding determined directly by the two stimuli presented in each trial (i.e. ∑Var and ΔVar), we also found choice-determined risk encoding expressed in greater activity when the chosen option was riskier than the surer option, as discussed further below. More broadly, the likelihood that multiple different aspects of risk are tracked within the brain during value-based choice is consistent with the phylogenetically ancient nature of risk sensitivity (Kacelnik and Bateson, 1996; Real et al., 1982) and its importance in decision-making (D' Acremont and Bossaerts, 2008).

With respect to EV, we found that encoding depends on which option was chosen (i.e. encoding of chosen and unchosen EVs), consistent with previous data reporting such encoding for SVs (FitzGerald et al., 2009; Rushworth et al., 2011). We observed this EV encoding in expected directions, with positive correlations for EV_chosen_ and negative correlations for EV_unchosen_ (Rushworth et al., 2011). We note that as well as identifying encoding in OFC, a region commonly associated with value (FitzGerald et al., 2009), we also observed activity in hippocampus, a region identified with reward processing in a meta-analysis of value-based choice (Liu et al., 2011), as well as in precuneus (Viard et al., 2011) a regions also implicated in goal-directed behaviour (Cavanna and Trimble, 2006). Further, because we report activity uniquely correlating with EV, we note that such EV encoding may be even more widespread as suggested by the marked activity seen in OFC, bilateral striatum and posterior cingulate for the contrast of gain relative to loss trials, where gain trials involve higher values.

EV encoding also depended upon whether outcomes were gains or losses. As here individuals must evaluate and select between two risky options of the same valence, our data suggest that valence may exert separable effects on chosen and unchosen values. Indeed, the pattern of this valence-dependent encoding highlights the importance of both chosen and unchosen values in shaping behaviour. We acknowledge that decisions are made in the context of only gains or losses in each trial, and future work could usefully examine situations where this was not the case, for example by mixing gains and losses. The greater activity seen here for gains relative to losses shows a striking commonality in striatum and vmPFC/OFC to that observed previously in a related task (Wright et al., 2012), consistent with the striatal expected value encoding seen in experiments using a mean–variance approach (Tobler et al., 2009).

In contrast to the highly robust neural data for summary statistic encoding, we did not find similarly clear evidence for encoding of SV over that of EV. However, we note that absence of evidence is not evidence of absence. Further, the large difference in value between the gain trials and loss trials may have led to more noise in valuation signals, which could have reduced the necessary sensitivity to identify parametric differences in EV or SV within trial types. With respect to reconciling our findings with those reporting integrated utility representations (Kable and Glimcher, 2007; Rangel et al., 2008), this raises a number of issues. Firstly, if choice is the product of multiple interacting decision systems, it may be that different tasks differentially involve different processes, such that some tasks induce model-based valuations approximating a unified utility signal, whilst others will not. Second, previous work may not always have conducted contrasts necessary to show neural data consistent with additional processes. For example with respect to valence, an interaction of choice and valence consistent with approach–avoidance was a central finding in DeMartino et al. (2006) as well as our previous dataset (Wright et al., 2012); but such a contrast was not reported in Tom et al. (2007) (see Wright et al., 2012 for more extensive discussion). Third, we note here that a recent study showing that SV representations of more complex multi-attribute stimuli may have a distributed representation, and may only be detectable using multivariate but not standard mass univariate analysis (Kahnt et al., 2011). Fourth, we note that EV representations here may in fact reflect model-based values, with the additional influence on action-selection from valence through distinct approach–avoidance processes that is not integrated within a unified utility signal.

Finally, how our manipulations of risk, valence and EV influence action-selection can be viewed from a perspective where choice is the product of multiple interacting decision systems that each influence action-selection, including both reflexive model-free systems, and more sophisticated model-based systems (Dayan, 2008). As in our previous Accept/Reject task (Wright et al., 2012) we find robust patterns of RTs and neural activity consistent with the hypothesis that risk and valence may, at least in part, influence action-selection through model-free approach–avoidance processes. Importantly, in model-free approach–avoidance processes the key feature is a contingency between stimulus properties and responsive actions — and we show these behavioural contingencies are selectively altered here in the Selection task relative to our previous Accept/Reject (Fig. 2), and that these selective changes are reflected in RT and neural data (Fig. 4). The RT findings here are explored and discussed in more detail in a series of previous related experiments (Wright et al., 2012). We note that whilst our design here precluded risk-related RT findings being explained by a motor habit (the side on which the riskier option appeared was random), future work could examine the possibility that they may relate to a higher level type of “habit” by dissociating the number of riskier choices from risk preference. Again, anterior insula is implicated here and this is a region known to be involved in representing aversive stimuli (Calder et al., 2001; Seymour et al., 2007), but has also been related to interoception (Critchley et al., 2004) and decision-making and addiction (Naqvi and Bechara, 2010). In model-based systems, stimulus features may be incorporated within a unified SV computed for each option, and action-selection involves choosing the option with the highest SV. We may not find clear evidence for unified SV encoding in addition to that for EV, but note that EV encoding itself may reflect a model-based computation and indeed that EV may influence action-selection through comparison of such model-based values.

Together these data are consistent with a biologically-based account of choice (Wright et al., 2012), where choice is a process involving both option-evaluation and action-selection (Corrado et al., 2009), and is likely to reflect the influence of multiple interacting decision systems (Dayan, 2008). Specifically, option evaluation may involve summary statistics. Action-selection may involve both model-based integration of summary statistics (EV and risk) that influence action-selection through comparator processes, and also model-free approach–avoidance responses to stimulus properties such as valence not requiring model-based processing.

In conclusion, our data support the suggestion that “summary statistics” describe the distribution of possible outcomes from a risky choice. Our data also show that in keeping with the importance of risk in decision-making, multiple aspects of risk are encoded during value-based choice, including both the sum and difference in risk between two risky options. Instead, neural data here suggested that EV encoding reflected chosen and unchosen EVs, and was also crucially dependent on outcome valence. Our data thus support a hypothesis that the brain encodes “summary statistics” describing the distribution of potential outcomes during risky choice, and highlight differences between the encoding of these summary statistics.

## Figures and Tables

**Table 1:** Correlations between stimulus aspects. By design there was no correlation between ΔEV and ΔVariance, although due to a limit in the number of trials this was not possible for all other stimulus aspects. In this table, r values are given and significant correlations are denoted by *.

	ΔVar	∑EV	∑Var
ΔEV	0.00	0.00	− 0.06
ΔVar		− 0.56*	0.88*
∑EV			− 0.6*

**Table 2:** fMRI factorial analysis. This table shows all activity from our main GLM surviving cluster level correction across the whole brain (P < 0.05 FWE corrected; voxel threshold of P < 0.005 used to define the clusters) for the specified contrasts. Panel a) shows results across subjects for the main effects of valence (gain versus loss) and choice (riskier versus surer) and their interactions. In addition to these whole brain corrected results, the contrast of losses > gains also revealed bilateral anterior insula activity (details in Results). Panel b) shows results between subjects using the second level covariates for risk (*PropRisk_all_*) or valence (*ImpValence*) for all main effects and interactions in our factorial design, and within each valence. For each cluster is shown: the three constituent peaks (> 8 mm apart) with the highest Z-scores; the number of voxels at P < 0.005 (unc.); and the P-value of the cluster after FWE correction across the whole brain (IFG = Inferior Frontal Gyrus: SMA = Supplementary Motor Area; dmPFC = dorsomedial PFC).

a) Across subjects							
Region	L/R	x	y	z	Z	#vox	p
*Gain* > *loss*							
IFG (p. Orb)	L	− 36	32	− 14	6.5	5106	0
Mid. orbital gyr.	L/R	− 6	50	− 11	6.2		
Amygdala	L	− 21	− 4	− 11	5.8		
Postr. cingulate cortex	L/R	− 3	− 49	19	5.5	528	< 1E-05
	− 6	− 49	31	5.1		
	9	− 40	31	4.1		
Angular gyr.	L	− 45	− 73	31	4.4	274	0.001
Mid. temporal gyr.		− 45	− 58	22	3.6		
	− 36	− 49	22	3		
Angular gyr.	R	51	− 64	25	3.9	148	0.033
	48	− 64	34	3.6		
	39	− 61	34	3.1		
Inf. parietal lobule	R	51	− 34	55	3.8	205	0.007
Supramarginal gyr.		63	− 19	28	3.6		
Postcentral gyr.		57	− 25	49	3.6		
Mid. temporal gyr.	R	57	− 58	− 2	3.7	156	0.026
	63	− 46	− 8	3.6		
Infr. temporal gyr.		48	− 46	− 5	3.2		

*Loss* > *gain*							
SMA	R	6	11	49	4.6	302	< 1E-03
	− 3	11	49	4.5		
Mid. frontal gyr.		− 27	− 1	55	3.7		

*Riskier* > *surer*							
Precuneus	R	21	− 73	40	4.2	615	< 1E-05
Supr. parietal lobule		21	− 55	55	4.1		
Angular gyr.		42	− 70	37	4		
MCC	L/R	− 6	17	37	4.2	1038	< 1E-08
dmPFC		15	62	22	4.1		
	9	50	31	3.9		
Antr. insula/IFG	R	51	17	1	4.1	284	0.001
	42	23	1	3.8		
Mid. temporal gyr.		54	2	− 23	3.3		
Cerebellum	L/R	3	− 49	− 8	4.1	1831	< 1E-13
Mid. orbital gyr.	L	− 48	− 79	7	3.9		
Infr. temporal gyr.	R	51	− 58	− 5	3.8		
Cerebellum	R	12	− 61	− 47	4.6	153	0.033
	6	− 55	− 41	4.5		
	− 6	− 49	− 44	3.7		
Cerebellum	L	− 27	− 58	− 44	4.3	225	0.005
	− 30	− 67	− 38	3.9		
	− 30	− 46	− 38	3.7		

b) Between subjects							

Region	L/R	x	y	z	Z	#vox	p

*PropRisk_all_* (*neg. correl*.) *on riskier* > *surer*							
Antr. insula	R	9	26	46	5.3	2035	< 1E-15
	30	20	10	4.9		
	45	17	− 2	4.7		
IFG (p. Orb.)	L	− 42	47	− 11	4.7	855	< 1E-07
IFG (p. Oper.)		− 51	14	4	4.4		
Antr. insula		− 27	26	1	4.4		
Postr. parietal cortex	R	33	− 43	40	5.0	4158	0
Occipital gyr.	L/R	− 42	− 82	− 5	4.9		
	15	− 73	7	4.8		
Thalamus	R	15	− 25	4	4.6	336	< 1E-03
	9	− 13	10	4.2		
	9	5	1	3.9		

*PropRisk_gain_* (*neg. correl*.) *on riskier* > *surer in gains*							
Mid. frontal gyr.	R	30	8	61	4.1	263	0.003
SMA		6	14	52	3.8		
	18	8	49	3.6		
Antr. insula	R	42	20	1	4.8	437	< 1E-04
	30	23	− 2	4.2		
Mid. frontal gyr.		48	41	19	3.7		
Postr. parietal lobule	R	30	− 49	37	4.4	348	< 1E-03
	30	− 58	43	4.2		
	39	− 40	40	3.7		
Postr. parietal lobule	L	− 48	− 40	43	4.0	732	< 1E-06
Occipital gyr.		− 36	− 85	− 8	4.7		
	− 24	− 67	34	4.2		

*PropRisk_loss_* (*neg. correl.*) *on riskier* > *surer in losses*							
preSMA	L/R	9	20	49	4.7	467	< 1E-04
SMA	9	11	55	4.2			
ACC	9	23	25	4.4			
Antr. insula	R	33	20	10	4.3	377	< 1E-03
	36	17	− 11	4.1		
	27	17	16	3.8		
Antr. insula	L	− 27	29	1	4.7	535	< 1E-05
	− 33	14	10	4.0		
Mid. frontal gyr.		− 39	47	19	3.8		
Postr. parietal lobule	L	− 48	− 49	43	3.9	338	< 1E-03
	− 33	− 43	34	3.9		
	− 24	− 61	49	3.8		
Infr. parietal lobule	R	36	− 43	37	3.8	161	0.029
	48	− 43	46	3.5		

**Table 3:** fMRI parametric analysis. This table shows all activity surviving cluster level correction across the whole brain (P < 0.05 FWE corrected; voxel threshold of P < 0.005 used to define the clusters) for the specified contrasts. Panel a) We test for stimulus-determined encoding in a new GLM with four parametric regressors: ∑EV, ΔEV, ∑Var, ΔVar. Panel b) We test choice-determined encoding in a further GLM with four parametric regressors: EV_chosen_, EV_unchosen_, V_archosen_, Var_unchosen_. In both new GLMs we do not orthogonalise these parametric regressors, which enable us to examine activity uniquely attributable to each regressor (i.e. not activity correlating with their shared components). Panel c) We test if encoding is valence-dependent by directly contrasting the parametric modulators in gains versus losses (and vice versa), shown here for all four parametric regressors in the choice-determined model (a similar pattern was seen in the stimulus determined model, Table S3). Note that for one subject who was very risk averse (i.e. few riskier choices) neither model could be estimated and thus for these models n = 24. For each cluster is shown: the three constituent peaks (> 8 mm apart) with the highest Z-scores; the number of voxels at P < 0.005 (unc.); and the P-value of the cluster after FWE correction across the whole brain (IFG = Inferior Frontal Gyrus: SMA = Supplementary Motor Area; dmPFC = dorsomedial PF; vmPFC = ventromedial PFC; ACC = Anterior Cingulate Cortex).

a) Stimulus-determined							
Region	L/R	x	y	z	Z	#vox	p
*ΣVar* (*pos. correl*.)							
Infr. parietal lobule	R	51	− 34	52	4.27	186	0.008
	36	− 43	55	3.48		
	30	− 49	55	3.25		
Infr. parietal lobule	L	− 33	− 67	40	4.18	132	0.041
Supr. parietal lobule		− 21	− 73	49	3.17		
IFG (p. Orb.)	L	− 48	47	− 8	4.69	695	< 1E-07
	− 51	38	− 5	4.69		
IFG (p. Tri.)		− 36	29	22	4.50		
IFG (p. Tri.)	R	54	20	16	4.21	713	< 1E-07
	51	29	31	4.14		
Mid. frontal gyr.		45	35	22	4.09		
Cerebellum	L	− 12	− 82	− 35	5.09	1540	< 1E-13
Fusiform gyr.	R	27	− 82	− 14	4.88		
Infr. temporal gyr.	L	− 48	− 58	− 5	4.68		

*∆Var* (*pos. correl*.)							
vmPFC/subgenual ACC	L/R	15	47	1	4.76	341	< 1E-03
	− 12	47	− 2	4.70		
	9	44	7	4.60		

*∆Var* (*neg. correl*.)							
IFG (p. Tri.)	R	57	23	22	4.02	348	< 1E-03
	48	29	25	4.00		
	51	38	16	3.50		
IFG (p. Tri.)	L	− 36	26	22	3.64	151	0.04
	− 54	17	28	4.05		
IFG (p. Oper.)		− 45	11	28	3.48		
Fusiform gyr.	R	27	− 79	− 14	4.31	1572	< 1E-11
Cerebellum		18	− 76	− 41	4.14		
Calcarine gyr.		30	− 64	7	4.09		

b) Choice-determined							

Region	L/R	x	y	z	Z	#vox	p

*Var_Chosen_* (*pos. correl*.)							
Mid. occipital gyr.	R	30	− 67	37	3.75	232	0.011
Angular gyr.		30	− 64	46	3.54		
Supr. parietal gyr.		24	− 76	49	3.11		

*EV_chosen_* (*pos. correl*.)							
Precuneus	L	− 12	− 52	64	3.78	154	0.024
	− 18	− 46	55	3.54		
	− 24	− 43	46	3.49		

*EV_unchosen_* (*neg. correl.*)							
Supr. temporal gyr.	L	− 63	− 22	7	4.04	161	0.035
Postcentral gyr.		− 63	− 7	19	3.95		
Supr. temporal gyr.		− 63	− 10	1	3.68		
Hippoc./parahippoc.	L	− 30	− 28	− 17	4.46	174	0.025
Fusiform gyr.		− 30	− 43	− 20	4.26		
Cerebellum		− 30	− 52	− 29	3.58		
Cerebellum	L	− 12	− 58	− 23	4.58	174	0.025
	3	− 58	− 29	3.37		
	− 15	− 55	− 38	3.05		

c) Choice-determined (interactions with valence)							

Region	L/R	x	y	z	Z	#vox	p

*EV_chosen_* (*gain* > *loss*)							
dmPFC	L/R	0	38	37	5.04	1795	< 1E-12
ACC	− 3	50	10	4.86			
Supr. frontal gyr.	R	21	56	34	4.77		
Antr. insula	L	− 48	26	4	4.02	217	0.008
	− 33	11	− 2	3.73		
	− 27	20	− 14	3.58		
Angular gyr.	L	− 45	− 64	40	4.52	225	0.006
Infer. parietal lobule		− 57	− 58	37	3.99		
Angular gyr.		− 42	− 55	34	3.62		
Mid. temporal gyr.	L	− 60	− 25	− 8	5.32	367	< 1E-03
	− 48	5	− 32	4.17		
	− 57	− 16	− 23	4.10		
Mid. temporal gyr.	R	63	− 22	− 14	5.04	369	< 1E-03
Infer. temporal gyr.		57	− 19	− 23	4.37		
	42	2	− 35	4.04		

*EV_unchosen_* (*loss* > *gain*)							
pre-SMA/SMA	L/R	9	8	49	4.07	391	< 1E-04
	0	11	52	3.82		
Mid. cingulate cortex	R	15	20	37	3.77		
Antr. insula/IFG	R	42	26	25	3.91	224	0.002
	33	23	10	3.53		
Putamen		30	8	13	3.48		
Insula	L	− 24	− 31	22	5.25	134	0.038
	− 21	− 16	28	3.69		
	− 33	14	13	3.40		
Supramarginal gyr.	R	63	− 25	19	3.30	135	0.036
Rolandic operculum		57	− 19	16	3.28		
Supramarginal gyr.		51	− 22	25	3.25		
Occipital	L/R	6	− 64	16	4.54	1536	< 1E-13
	− 33	− 76	28	4.11		
Supr. parietal lobule	R	24	− 64	55	4.02		

## References

[bb0005] Bossaerts P. (2010). Risk and risk prediction error signals in anterior insula. Brain Struct. Funct..

[bb0010] Calder A.J., Lawrence A.D., Young A.W. (2001). Neuropsychology of fear and loathing. Nat. Rev. Neurosci..

[bb0015] Cavanna A.E., Trimble M.R. (2006). The precuneus: a review of its functional anatomy and behavioural correlates. Brain.

[bb0020] Corrado G.S., Sugrue L.P., Brown J.R., Newsome W.T. (2009). The trouble with choice: studying decision variables in the brain. Neuroeconomics: Decision Making and the Brain.

[bb9000] Critchley H.D., Wiens S., Rotshtein P., Ohman A., Dolan R.J. (2004). Neural systems supporting interoceptive awareness. Nat. Neurosci..

[bb0025] D' Acremont M., Bossaerts P. (2008). Neurobiological studies of risk assessment: a comparison of expected utility and mean–variance approaches. Cogn. Affect. Behav. Neurosci..

[bb0030] Dayan P. (2008). The role of value systems in decision making. Better Than Conscious.

[bb9005] De Martino B., Kumaran D., Seymour B.J., Dolan R.J. (2006). Frames, biases, and rational decision-making in the human brain. Science.

[bb0035] FitzGerald T.H.B., Seymour B., Dolan R.J. (2009). The role of human orbitofrontal cortex in value comparison for incommensurable objects. J. Neurosci..

[bb9020] Friston K.J., Worsley K.J., Frackowiak R.S.J., Mazziotta J.C., Evans A.C. (1993). Assessing the significance of focal activations using their spatial extent. Human Brain Mapping.

[bb0040] Friston K.J., Frackowiak R.S.J. (2004). Experimental design and statistical parametric mapping. Human Brain Function.

[bb0045] Guitart-Masip M., Fuentemilla L., Bach D.R., Huys Q.J.M., Dayan P., Dolan R.J., Duzel E. (2011). Action dominates valence in anticipatory representations in the human striatum and dopaminergic midbrain. J. Neurosci..

[bb0050] Huettel S.A., Song A.W., McCarthy G. (2005). Decisions under uncertainty: probabilistic context influences activation of prefrontal and parietal cortices. J. Neurosci..

[bb0055] Kable J.W., Glimcher P.W. (2007). The neural correlates of subjective value during intertemporal choice. Nat. Neurosci..

[bb0060] Kacelnik A., Bateson M. (1996). Risky theories—the effects of variance on foraging decisions. Am. Zool..

[bb0065] Kahneman D., Tversky A. (1979). Prospect theory: an analysis of decision under risk. Econometrica.

[bb0070] Kahnt T., Heinzle J., Park S.Q., Haynes J.-D. (2011). Decoding different roles for vmPFC and dlPFC in multi-attribute decision making. Neuroimage.

[bb0075] Liu X., Hairston J., Schrier M., Fan J. (2011). Common and distinct networks underlying reward valence and processing stages: a meta-analysis of functional neuroimaging studies. Neurosci. Biobehav. Rev..

[bb0080] Mohr P.N.C., Biele G., Heekeren H.R. (2010). Neural processing of risk. J. Neurosci..

[bb9010] Naqvi N.H., Bechara A. (2010). The insula and drug addiction: an interoceptive view of pleasure, urges, and decision-making. Brain Struct Funct.

[bb9015] O'Doherty J., Dayan P., Schultz J., Deichmann R., Friston K., Dolan R.J. (2004). Dissociable roles of ventral and dorsal striatum in instrumental conditioning. Science.

[bb0085] Platt M.L., Glimcher P.W. (1999). Neural correlates of decision variables in parietal cortex. Nature.

[bb0090] Preuschoff K., Bossaerts P., Quartz S. (2006). Neural differentiation of expected reward and risk in human subcortical structures. Neuron.

[bb0095] Rangel A., Camerer C., Montague P.R. (2008). A framework for studying the neurobiology of value-based decision making. Nat. Rev. Neurosci..

[bb0100] Real L., Ott J., Silverfine E. (1982). On the tradeoff between the mean and the variance in foraging: effect of spatial distribution and color preference. Ecology.

[bb0105] Rushworth M.F.S., Noonan M.P., Boorman E.D., Walton M.E., Behrens T.E. (2011). Frontal cortex and reward-guided learning and decision-making. Neuron.

[bb0110] Schwarz G. (1978). Estimating the dimension of a model. Ann. Stat..

[bb0115] Seymour B.J., Singer T., Dolan R. (2007). The neurobiology of punishment. Nat. Rev. Neurosci..

[bb0120] Tobler P.N., Christopoulos G.I., O'Doherty J.P., Dolan R.J., Schultz W. (2009). Risk-dependent reward value signal in human prefrontal cortex. Proc. Natl. Acad. Sci..

[bb8000] Tom S.M., Fox C.R., Trepel C., Poldrack R.A. (2007). The neural basis of loss aversion in decision-making under risk. Science.

[bb0125] Viard A., Doeller C.F., Hartley T., Bird C.M., Burgess N. (2011). Anterior hippocampus and goal-directed spatial decision making. J. Neurosci..

[bb0130] Wright N.D., Symmonds M., Hodgson K., Fitzgerald T.H.B., Crawford B., Dolan R.J. (2012). Approach–avoidance processes contribute to dissociable impacts of risk and loss on choice. J. Neurosci..

