# Supplementary material for: Distinct encoding of risk and value in economic choice between multiple risky options
Source: Neuroimage. 2013 Nov 1;81:431–40. doi: 10.1016/j.neuroimage.2013.05.023 (PMC3734351; doi:10.1016/j.neuroimage.2013.05.023)
Supplement: Supplementary file 1 — Supplementary material. [file mmc1.docx]

# Supplementary materials: Distinct encoding of risk and value in economic choice between multiple risky options

Nicholas D Wright, Mkael Symmonds, Raymond J Dolan

## Supplementary results

#### Additional Factorial fMRI analyses

**Approaching riskier relative to surer options:** Regarding risk, across subjects behaviourally there was the same aversion to risk overall (i.e. collapsed across valence) as in our previous task (Fig. 2), and this similarity was reflected neurally. There was increased activity for approaching (choosing) the riskier relative to the surer option in multiple regions (right parietal cortex, mid-cingulate/dmPFC, right anterior insula/IFG); but nothing for the reverse contrast (i.e. chose surer>riskier). This asymmetry is illustrated by the number of suprathreshold voxels (at P<0.005 unc.) across the brain for these contrasts, with 4977 for the former but only 35 for the reverse. Furthermore, as in our previous task, between subjects our data were also strikingly asymmetric and consistent with an approach-avoidance hypothesis (Fig. 4). Here the more averse an individual was to risk (i.e. lower PropRisk_all_), the greater the activity evoked when approaching (choosing) the riskier option in areas including bilateral anterior insula, dmPFC/SMA, bilateral parietal cortex and thalamus/caudate. Again for illustration there were 7690 suprathreshold voxels (P<0.005 unc.) for that correlation but only 52 for the reverse.

Testing activity for approaching risk in each valence separately also revealed strikingly asymmetric patterns across and between subjects. Across subjects, again revealed whole brain corrected activity for riskier>surer choices with both gains (including posterior parietal, pre-SMA/dmPFC, left IFG; Table S2) and losses (subgenual ACC/vmPFC whole brain corrected and left nucleus accumbens SVC), but nothing for the reverse contrasts (only 8 suprathreshold voxels with losses at P<0.005 and only 25 voxels with gains). Between subjects, the more averse an individual was to risk in gains (i.e. lower PropRisk_gain_), the greater the activity for approaching the riskier than surer option in gain trials (including right anterior insula, SMA/ACC and right parietal cortex) with nothing seen for the reverse. Very similar areas showed a correlation between PropRisk_loss_ and activity for the contrast of riskier>surer choices in loss trials (including bilateral anterior insula, SMA/ACC and bilateral parietal cortex), with nothing for the reverse. We also note that no activity was seen in any of these regions when correlating PropRisk_gain_ with activity for riskier>surer choices in loss trials, or PropRisk_loss_ with such activity in gain trials.

**Alternative factorial designs:** We next asked whether such activity is specific to choosing (approaching) the riskier relative to the surer option, or occurs more generally when choosing options containing less preferred stimulus aspects (e.g. lower EV or lower SV). First, we asked if choosing the lower EV versus higher EV option in each trial led to similar neural activity, using an alternative factorial design (2 valence [gain, loss] by 2 choice [higherEV, lowerEV], with parametric regressors as before). Unlike in our main model, neither for the contrast of choosing the lower EV than higher EV option, nor the reverse, did activity survive whole brain correction (although we note the same robust activity for gains>losses and the reverse; Table S1). Second, we used subject-specific SVs from the winning behavioural model to define choice (i.e. as higher SV and lower SV). However, a potential confound here is that when risk-averse the lower SV option also tends to be the riskier option (and vice versa when risk-seeking). Therefore, we estimated one model with two onset regressors (gains, losses) and four orthogonalised parametric regressors (lower or higher SV choice [as 0 or 1]; lower or higher risk choice [as 0 or 1]; ΔEV; ΔVar], and an alternative model with the order of the two choice regressors reversed. Whilst the model with lower/higher SV first shows greater activity for lower than higher SV choices in anterior insula and SMA, this is removed by putting the riskier/surer choice regressor first (i.e. by removing activity correlated with approaching the riskier option). However, we note that even when putting either a lower/higher SV regressor or a lower/higher EV choice regressor first before the riskier/surer choice regressor, we still see widespread whole brain corrected activity for riskier/surer choice (Table S1).

## Supplementary Figures


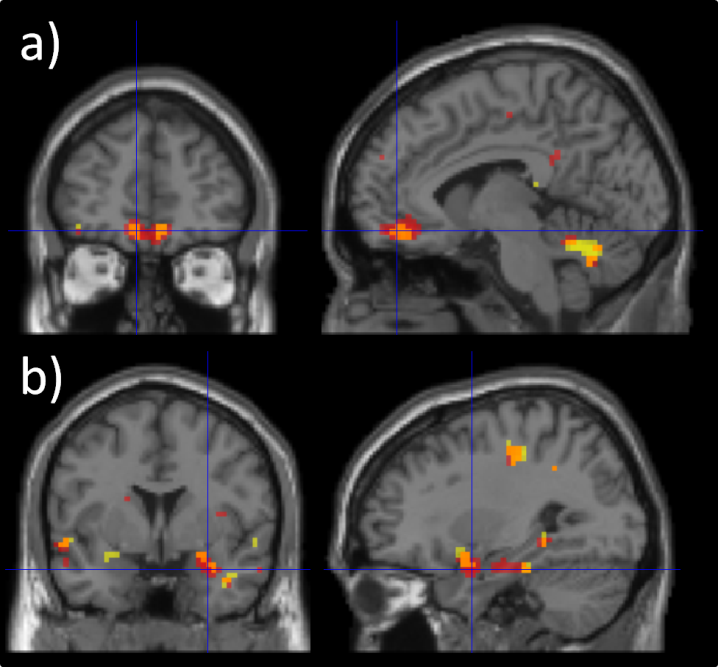


**Supplementary Figure 1 Overlap of SV and EV related activity.** In those regions correlating with SV, activity is also seen for EV. Here is shown activity correlating with SVunchosen in OFC (panel a) and right amygdala (panel b) in red and EVunchosen in yellow. Shown at P<0.005 uncorrected, at the peak coordinates in each region for SVunchosen (see Table S4 for details).

**Supplementary Figure 2 Plot of ∑Var and ∑EV in the set of 100 trials.** See Table 1 for correlation between these measures (r=-0.6, P<0.05).

## Supplementary Tables


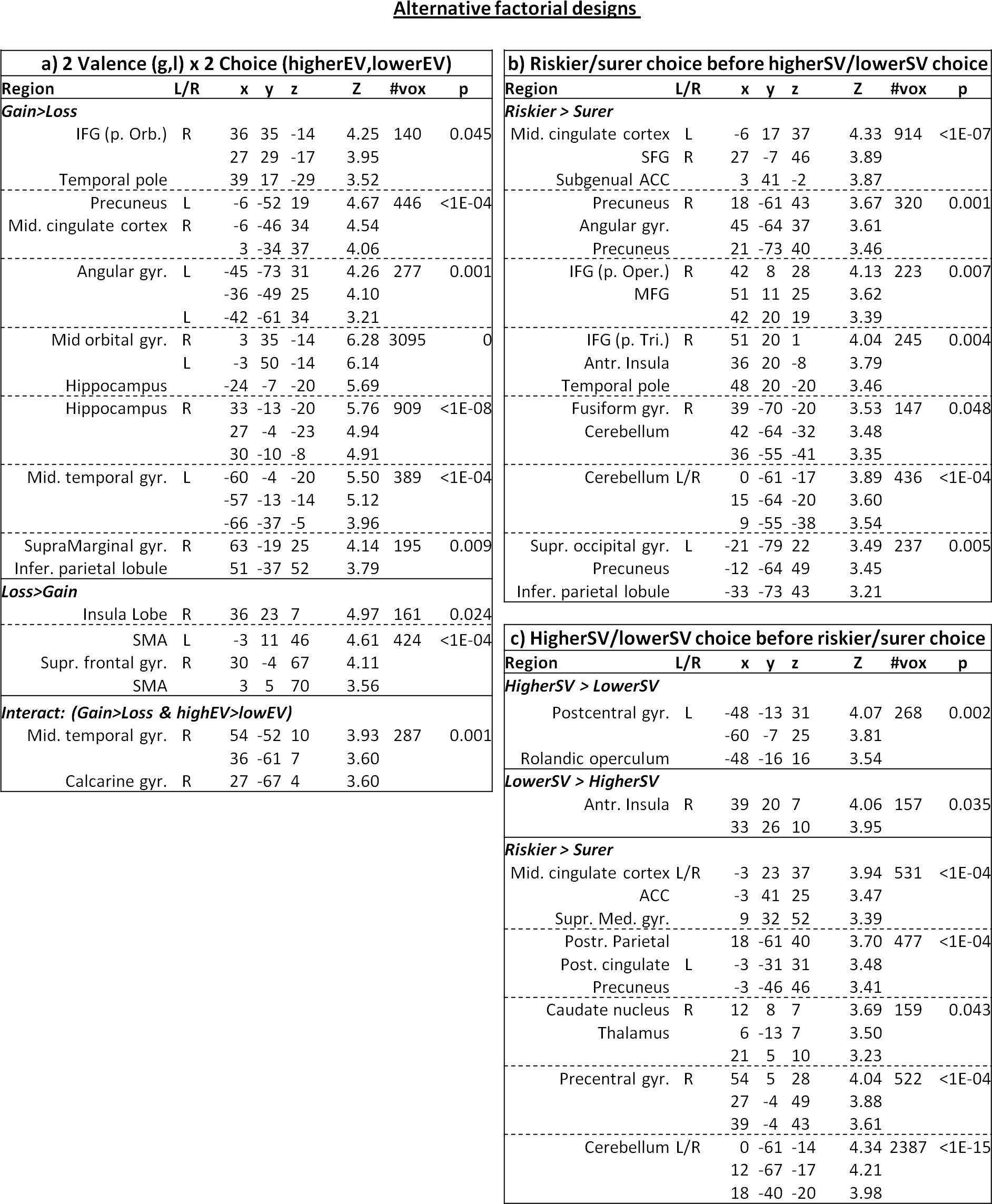


**Supplementary Table 1 Alternative factorial designs.** We asked whether increased activity is specific to choosing (approaching) the riskier relative to the surer option, or occurs more generally when choosing options containing less preferred stimulus aspects (e.g. lower EV or lower SV). This table shows all activity surviving cluster level correction across the whole brain (P<0.05 FWE corrected; voxel threshold of P<0.005 used to define the clusters) for the contrasts specified. First, in **panel** **a)** we address if choosing the lower EV versus higher EV option in each trial led to similar neural activity, reporting an alternative factorial design (2 valence [gain, loss] by 2 choice [higherEV, lowerEV], with parametric regressors as before). We test for the main effects of valence (gain versus loss) and choice (higherEV versus lowerEV) and their interactions. Second, we used subject-specific SVs from the winning behavioural model to define choice (i.e. as higher SV and lower SV). However, a potential confound here is that when risk-averse the lower SV option also tends to be the riskier option (and vice versa when risk-seeking). Therefore, shown in **panel b)** we estimated one model with two onset regressors (gains, losses) and four orthogonalised parametric regressors (lower or higher risk choice [as 0 or 1]; lower or higher SV choice [as 0 or 1]; ΔEV; ΔVar] – and this reveals the expected activity for riskier>surer choices and no activity related to higher/lower SV choices. In **panel c)** we show that in an alternative model with the order of the two choice regressors reversed, activity for riskier>surer choices survives the removal of the shared component with the higher/lower SV choice regressor. In addition, in the model reported in panel c), activity for riskier>surer choices in bilateral anterior insula also survived small volume correction (Left: -33 17 8; 47 vox; Z 3.2; Right: 33 17 -5; 121 vox; Z 3.4). (IFG = Inferior Frontal Gyrus; SMA = Supplementary Motor Area; SFG = Superior Frontal Gyrus; MFG = Medial Frontal Gyrus; ACC = Anterior Cingulate Cortex).


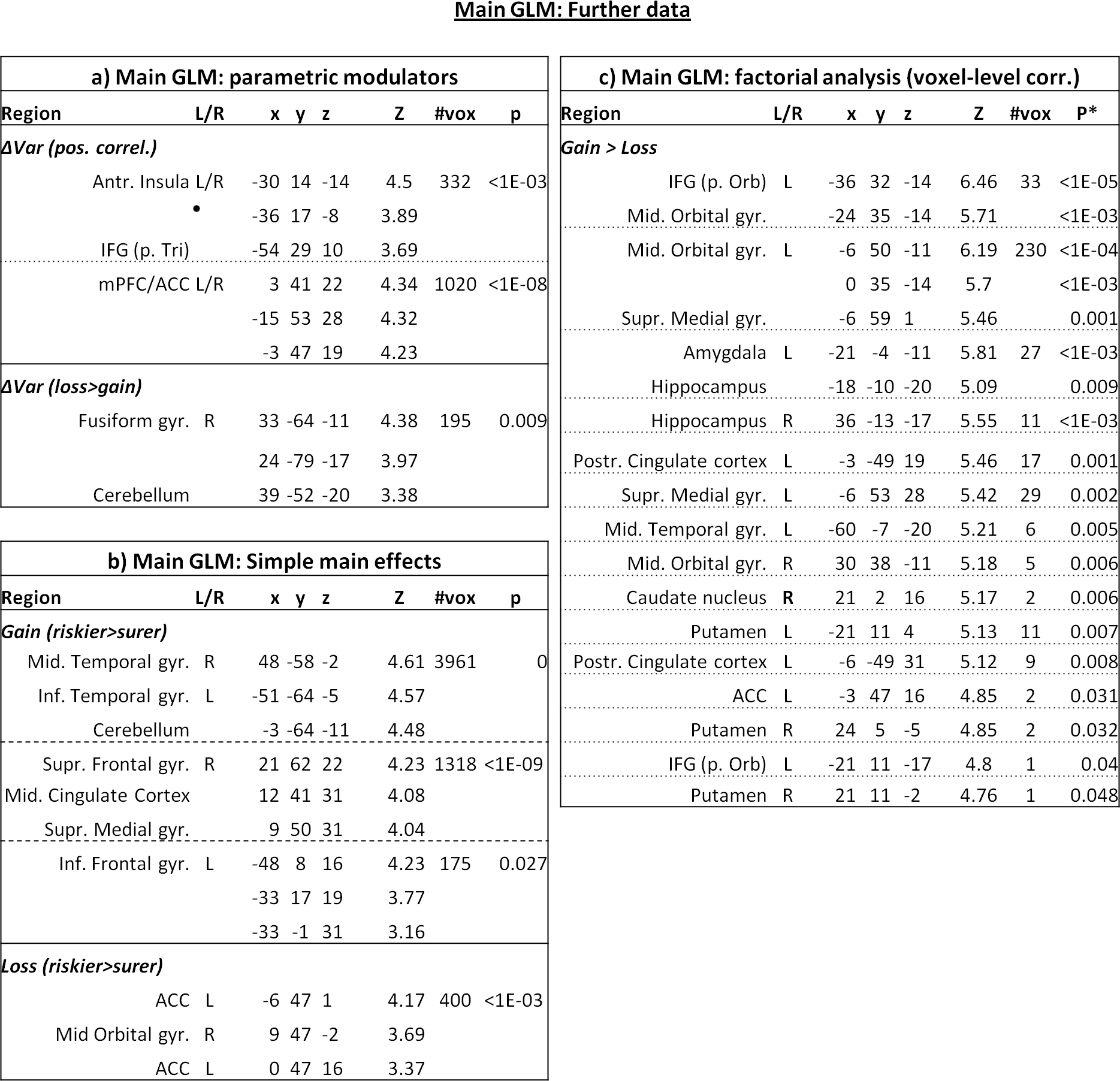


**Supplementary Table 2 Additional contrasts from the main GLM.** The main GLM used a 2 valence (gain, loss) by 2 choice (riskier, surer) design with parametric modulators of ΔVar and ΔEV. This table shows activity surviving cluster level correction across the whole brain (P<0.05 FWE corrected; voxel threshold of P<0.005 used to define the clusters) for the contrasts specified, that for reasons of space were not included in Table 2. **Panel** **a)** shows activity for the parametric modulators ΔVar and ΔEV (positive and negative correlations and interactions with valence for each regressor). **Panel b)** We test for simple main effects of riskier versus surer choices in each valence. **Panel c)** To identify individual areas within the large clusters seen for the contrast of gains>losses in Table 2, here use a more stringent threshold of FWE correction at the voxel-level (P<0.05) and report all main effects and interactions surviving that threshold (p* here is FWE corrected at the voxel level). (IFG = Inferior Frontal Gyrus; mPFC = medial PFC; ACC = Anterior Cingulate Cortex).


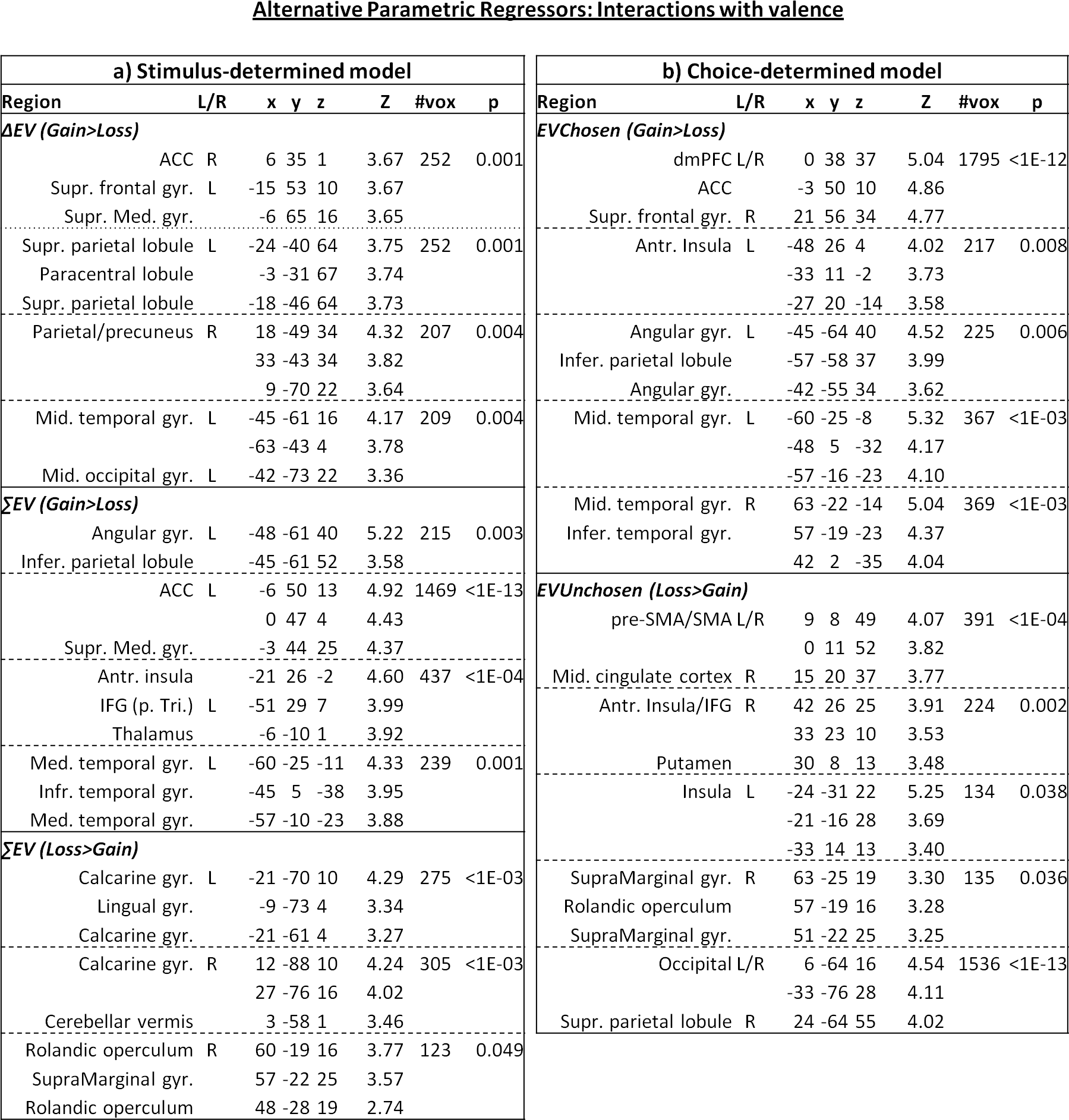


**Supplementary Table 3 Interaction of EV and risk encoding with valence.** This table shows activity surviving cluster level correction across the whole brain (P<0.05 FWE corrected; voxel threshold of P<0.005 used to define the clusters) for the contrasts specified. We tested for interactions of parametric regressors with valence, by contrasting them in gains>losses and losses>gains. Similar results are seen in the GLM with stimulus-determined parametric regressors (∑EV, ΔEV, ∑Var, ΔVar) shown in **panel a)** and also in the GLM with choice-determined encoding (EV_chosen_, EV_unchosen_, Var_chosen_, Var_unchosen_) shown in **panel b**.


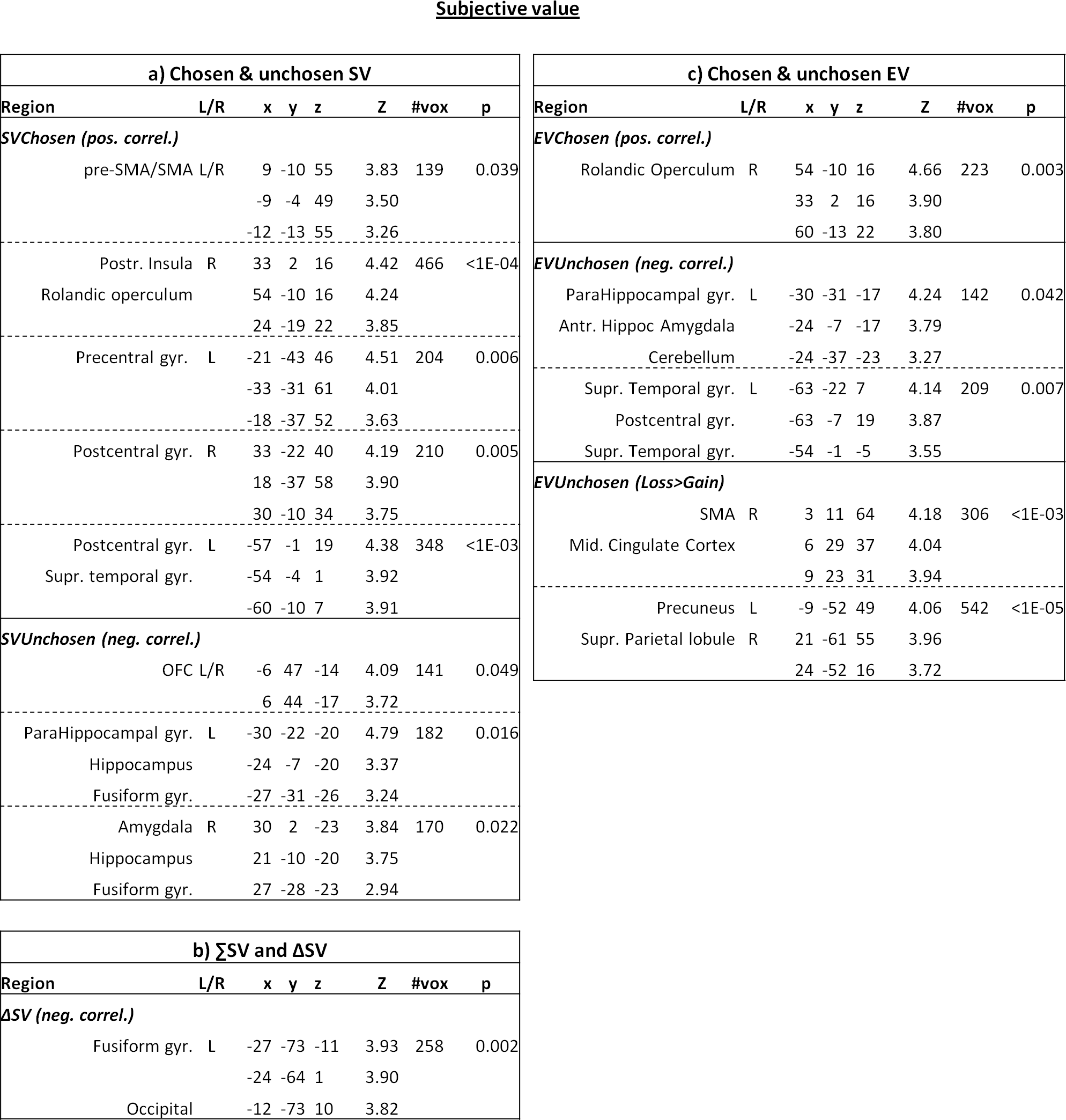


**Supplementary Table 4 Subjective value.** This table shows activity surviving cluster level correction across the whole brain (P<0.05 FWE corrected; voxel threshold of P<0.005 used to define the clusters) for the contrasts specified. Activity is shown correlating with parametric regressors (positive and negative correlations or interactions with valence) in the following three models adapted from our main GLM: **a)** with parametric regresssors of SV_Chosen_ and SV_Unchosen_; **b)** with parametric regressors of ∑SV and ΔSV; and **c)** with parametric regressors of EV_Chosen_ and EV_Unchosen_.

| Lottery 1 | | | | Lottery 2 | | | |
| --- | --- | --- | --- | --- | --- | --- | --- |
| p1 | p2 | a1 | a2 | p1 | p2 | a1 | a2 |
| 0.25 | 0.75 | 2 | 24 | 0.25 | 0.75 | 12 | 22 |
| 0.25 | 0.75 | 10 | 20 | 0.25 | 0.75 | 2 | 24 |
| 0.35 | 0.65 | 6 | 24 | 0.85 | 0.15 | 20 | 22 |
| 0.25 | 0.75 | 8 | 22 | 0.35 | 0.65 | 2 | 24 |
| 0.45 | 0.55 | 8 | 24 | 0.45 | 0.55 | 18 | 22 |
| 0.85 | 0.15 | 18 | 22 | 0.25 | 0.75 | 16 | 24 |
| 0.75 | 0.25 | 16 | 20 | 0.15 | 0.85 | 0 | 24 |
| 0.15 | 0.85 | 10 | 22 | 0.15 | 0.85 | 0 | 24 |
| 0.85 | 0.15 | 16 | 22 | 0.25 | 0.75 | 6 | 24 |
| 0.45 | 0.55 | 16 | 22 | 0.55 | 0.45 | 10 | 24 |
| 0.25 | 0.75 | 20 | 22 | 0.55 | 0.45 | 14 | 24 |
| 0.55 | 0.45 | 16 | 20 | 0.15 | 0.85 | 0 | 24 |
| 0.45 | 0.55 | 20 | 22 | 0.45 | 0.55 | 18 | 24 |
| 0.35 | 0.65 | 8 | 24 | 0.25 | 0.75 | 18 | 22 |
| 0.15 | 0.85 | 16 | 24 | 0.75 | 0.25 | 18 | 22 |
| 0.45 | 0.55 | 14 | 24 | 0.75 | 0.25 | 20 | 22 |
| 0.75 | 0.25 | 16 | 24 | 0.55 | 0.45 | 18 | 22 |
| 0.15 | 0.85 | 0 | 24 | 0.15 | 0.85 | 14 | 18 |
| 0.15 | 0.85 | 0 | 24 | 0.15 | 0.85 | 8 | 20 |
| 0.55 | 0.45 | 14 | 24 | 0.65 | 0.35 | 18 | 20 |
| 0.15 | 0.85 | 6 | 24 | 0.25 | 0.75 | 16 | 18 |
| 0.25 | 0.75 | 18 | 22 | 0.75 | 0.25 | 16 | 24 |
| 0.25 | 0.75 | 18 | 20 | 0.15 | 0.85 | 6 | 24 |
| 0.15 | 0.85 | 6 | 24 | 0.85 | 0.15 | 18 | 20 |
| 0.45 | 0.55 | 18 | 20 | 0.55 | 0.45 | 14 | 24 |
| 0.55 | 0.45 | 18 | 24 | 0.15 | 0.85 | 20 | 22 |
| 0.15 | 0.85 | 18 | 22 | 0.35 | 0.65 | 8 | 24 |
| 0.65 | 0.35 | 16 | 18 | 0.55 | 0.45 | 14 | 24 |
| 0.55 | 0.45 | 14 | 24 | 0.45 | 0.55 | 20 | 22 |
| 0.25 | 0.75 | 20 | 22 | 0.35 | 0.65 | 6 | 24 |
| 0.15 | 0.85 | 6 | 24 | 0.25 | 0.75 | 10 | 20 |
| 0.15 | 0.85 | 14 | 18 | 0.35 | 0.65 | 8 | 24 |
| 0.65 | 0.35 | 20 | 22 | 0.55 | 0.45 | 14 | 24 |
| 0.45 | 0.55 | 18 | 20 | 0.35 | 0.65 | 6 | 24 |
| 0.35 | 0.65 | 8 | 24 | 0.85 | 0.15 | 18 | 22 |
| 0.35 | 0.65 | 8 | 24 | 0.15 | 0.85 | 16 | 20 |
| 0.55 | 0.45 | 18 | 22 | 0.35 | 0.65 | 8 | 24 |
| 0.35 | 0.65 | 18 | 22 | 0.45 | 0.55 | 8 | 24 |
| 0.75 | 0.25 | 18 | 20 | 0.45 | 0.55 | 14 | 24 |
| 0.25 | 0.75 | 6 | 24 | 0.25 | 0.75 | 12 | 18 |
| 0.85 | 0.15 | 16 | 20 | 0.35 | 0.65 | 8 | 24 |
| 0.35 | 0.65 | 18 | 20 | 0.45 | 0.55 | 14 | 24 |
| 0.25 | 0.75 | 14 | 22 | 0.55 | 0.45 | 10 | 24 |
| 0.65 | 0.35 | 18 | 22 | 0.25 | 0.75 | 16 | 24 |
| 0.85 | 0.15 | 20 | 22 | 0.45 | 0.55 | 18 | 24 |
| 0.25 | 0.75 | 16 | 22 | 0.35 | 0.65 | 10 | 24 |
| 0.55 | 0.45 | 14 | 20 | 0.25 | 0.75 | 8 | 24 |
| 0.55 | 0.45 | 14 | 20 | 0.45 | 0.55 | 10 | 24 |
| 0.45 | 0.55 | 20 | 22 | 0.15 | 0.85 | 6 | 24 |
| 0.15 | 0.85 | 0 | 24 | 0.85 | 0.15 | 16 | 20 |
| 0.35 | 0.65 | 2 | 24 | 0.25 | 0.75 | 6 | 20 |
| 0.35 | 0.65 | 6 | 24 | 0.45 | 0.55 | 20 | 22 |
| 0.45 | 0.55 | 10 | 24 | 0.35 | 0.65 | 14 | 20 |
| 0.75 | 0.25 | 16 | 24 | 0.15 | 0.85 | 18 | 22 |
| 0.35 | 0.65 | 10 | 24 | 0.55 | 0.45 | 16 | 20 |
| 0.25 | 0.75 | 0 | 24 | 0.45 | 0.55 | 10 | 22 |
| 0.65 | 0.35 | 14 | 24 | 0.55 | 0.45 | 20 | 22 |
| 0.45 | 0.55 | 10 | 24 | 0.15 | 0.85 | 16 | 22 |
| 0.75 | 0.25 | 18 | 20 | 0.15 | 0.85 | 10 | 24 |
| 0.65 | 0.35 | 18 | 22 | 0.75 | 0.25 | 16 | 24 |
| 0.15 | 0.85 | 6 | 24 | 0.65 | 0.35 | 20 | 22 |
| 0.45 | 0.55 | 14 | 24 | 0.35 | 0.65 | 16 | 18 |
| 0.85 | 0.15 | 18 | 20 | 0.35 | 0.65 | 6 | 24 |
| 0.45 | 0.55 | 18 | 22 | 0.25 | 0.75 | 16 | 24 |
| 0.45 | 0.55 | 14 | 24 | 0.75 | 0.25 | 16 | 18 |
| 0.45 | 0.55 | 14 | 20 | 0.15 | 0.85 | 2 | 24 |
| 0.75 | 0.25 | 18 | 22 | 0.35 | 0.65 | 8 | 24 |
| 0.85 | 0.15 | 18 | 22 | 0.15 | 0.85 | 0 | 24 |
| 0.35 | 0.65 | 20 | 22 | 0.55 | 0.45 | 18 | 24 |
| 0.35 | 0.65 | 16 | 22 | 0.45 | 0.55 | 10 | 24 |
| 0.35 | 0.65 | 18 | 22 | 0.35 | 0.65 | 8 | 24 |
| 0.25 | 0.75 | 16 | 24 | 0.55 | 0.45 | 18 | 22 |
| 0.65 | 0.35 | 20 | 22 | 0.35 | 0.65 | 6 | 24 |
| 0.25 | 0.75 | 16 | 24 | 0.75 | 0.25 | 18 | 22 |
| 0.35 | 0.65 | 6 | 24 | 0.25 | 0.75 | 12 | 22 |
| 0.75 | 0.25 | 16 | 24 | 0.35 | 0.65 | 18 | 22 |
| 0.35 | 0.65 | 6 | 24 | 0.25 | 0.75 | 18 | 20 |
| 0.35 | 0.65 | 8 | 24 | 0.75 | 0.25 | 16 | 20 |
| 0.15 | 0.85 | 0 | 24 | 0.45 | 0.55 | 16 | 20 |
| 0.15 | 0.85 | 2 | 24 | 0.85 | 0.15 | 16 | 22 |
| 0.25 | 0.75 | 18 | 22 | 0.85 | 0.15 | 16 | 24 |
| 0.45 | 0.55 | 18 | 24 | 0.65 | 0.35 | 20 | 22 |
| 0.35 | 0.65 | 10 | 24 | 0.85 | 0.15 | 16 | 22 |
| 0.15 | 0.85 | 14 | 24 | 0.15 | 0.85 | 16 | 22 |
| 0.15 | 0.85 | 6 | 22 | 0.25 | 0.75 | 4 | 24 |
| 0.35 | 0.65 | 8 | 24 | 0.55 | 0.45 | 16 | 20 |
| 0.65 | 0.35 | 18 | 20 | 0.15 | 0.85 | 6 | 24 |
| 0.15 | 0.85 | 10 | 24 | 0.35 | 0.65 | 20 | 22 |
| 0.55 | 0.45 | 20 | 22 | 0.45 | 0.55 | 14 | 24 |
| 0.45 | 0.55 | 14 | 24 | 0.35 | 0.65 | 20 | 22 |
| 0.15 | 0.85 | 10 | 24 | 0.75 | 0.25 | 20 | 22 |
| 0.35 | 0.65 | 20 | 22 | 0.65 | 0.35 | 14 | 24 |
| 0.55 | 0.45 | 10 | 24 | 0.15 | 0.85 | 12 | 20 |
| 0.55 | 0.45 | 18 | 24 | 0.55 | 0.45 | 20 | 22 |
| 0.35 | 0.65 | 8 | 24 | 0.15 | 0.85 | 10 | 22 |
| 0.35 | 0.65 | 18 | 20 | 0.15 | 0.85 | 10 | 24 |
| 0.25 | 0.75 | 16 | 18 | 0.35 | 0.65 | 6 | 24 |
| 0.45 | 0.55 | 18 | 22 | 0.75 | 0.25 | 16 | 24 |
| 0.45 | 0.55 | 10 | 24 | 0.55 | 0.45 | 16 | 22 |
| 0.35 | 0.65 | 6 | 24 | 0.45 | 0.55 | 16 | 18 |

**Supplementary Table 5** Set of all amounts (a) and probabilities (p) used in each of the set of 100 gain trials.
